# Association of Handgrip Strength with Dietary Intake in the Korean Population: Findings Based on the Seventh Korea National Health and Nutrition Examination Survey (KNHANES VII-1), 2016

**DOI:** 10.3390/nu10091180

**Published:** 2018-08-28

**Authors:** Young Jin Tak, Jeong Gyu Lee, Yu Hyeon Yi, Yun Jin Kim, Sangyeoup Lee, Byung Mann Cho, Young Hye Cho

**Affiliations:** 1 Department of Family Medicine, Pusan National University Hospital, Busan 602-739, Korea; 03141998@hanmail.net (Y.J.T.); eeugus@gmail.com (Y.H.Y.); yujkim@pusan.ac.kr (Y.J.K.); 2 Medical Research Institute, Pusan National University Hospital, Busan 602-739, Korea; 3 Department of Family Medicine, Pusan National University School of Medicine, Busandaehak-ro, Mulgeum-eup, Yangsan-si 50612, Korea; younghye82@naver.com; 4 Busan Tobacco Control Center, Pusan National University Hospital, Busan 49241, Korea; 5 Department of Medical Education, Pusan National University School of Medicine, Busandaehak-ro, Mulgeum-eup, Yangsan-si 50612, Korea; saylee@pnu.edu; 6 Family Medicine Clinic, Pusan National University Yangsan Hospital, Geumo-ro, Mulgeum-eup, Yangsan-si 50612, Korea; 7 Department of Preventive Medicine and Occupational Medicine, Pusan National University School of Medicine, Busandaehak-ro, Mulgeum-eup, Yangsan-si 50612, Korea; bmcho@hanmail.net

**Keywords:** handgrip strength, polyunsaturated fatty acid, vitamin C, age, dietary fiber

## Abstract

To evaluate associations between handgrip strength (HGS) and dietary nutrients, this study of a representative Korean population of 1553 adults aged ≥60 years (706 men and 847 women) analyzed data from the Korea National Health and Nutrition Examination Survey (2016). HGS was measured in both hands three times using a digital grip strength dynamometer. Dietary intake data were collected by the 24-h recall method through computer-assisted personal interviews. The study population had a mean age of 70.1 years, body mass index (BMI) of 24.2 kg/m^2^, and HGS of 35.7 kg in men, 21.2 kg in women. Total energy (*r* = 0.411), protein (*r* = 0.217), polyunsaturated fatty acid (PUFA) (*r* = 0.269), fiber (*r* = 0.272), and vitamin C (*r* = 0.098) were positively correlated with HGS. In multivariable regression analysis, PUFA (*β* = 0.083) and vitamin C (*β* = 0.003) were positively associated with HGS among women. Fiber (*β* = 0.071) and vitamin C (*β* = 0.006) showed a positive association with HGS among men. Community-dwelling older men and women with higher levels of PUFA, fiber, and vitamin C in their diet were more likely to have greater HGS even after adjusting for age, total calorie intake, BMI, chronic diseases and health-related habits.

## 1. Introduction

Aging is the leading risk factor for functional decline, which can have serious health consequences, including loss of independence, hospitalization, and mortality [1]. Physical performance in older adults is considered a critical marker of actual biological age. Muscle strength is one of the most decisive indicators of health status in older adults [2]. Reduced muscle strength and power are closely associated with adverse health-related outcomes, including loss of independence, increased risk of mobility disability, and mortality (4% increase with every 1 kg decrease in grip strength) [3]. Several quantitative measurements are used to assess progressive decline in muscle strength in older adults, including handgrip strength (HGS) and the Timed Up-and-Go test [2,4]. HGS is the maximum static force applied by the hand, as measured by a dynamometer. As it is both simple and inexpensive to evaluate, this measurement is widely applied not only to assess hand function after injury and the outcome of hand surgery, but also to reflect general physical health and disability [5,6]. A previous longitudinal study found that poor HGS was related to increased mortality risk from cardiovascular disease and cancer, even after adjusting for body composition, multiple chronic diseases and multi-morbidity, in both men and women [7].

Healthy dietary habits are crucial to the maintenance of physical health in later life. Undernutrition is often observed in older adults [8], Several nutritional evaluation tools are used for early identification of people at risk of undernutrition. Among the most commonly applied and reliable assessment methods for this purpose is 24-h dietary recall, which constitutes a logical approach to examining the nutrients that influence muscle strength and the underlying mechanisms, because it captures actual food consumption. In addition, recent studies imply that HGS can be used as a predictor of nutritional status [9,10].

Several human studies have attempted to identify the relationships of nutrient intakes with sarcopenic changes and muscle strength. The majority of nutrition-related observational or interventional studies have investigated the effects on muscle strength of single macronutrients (e.g., protein) and micronutrients (e.g., vitamin B12, vitamin D, antioxidants). However, these studies had major limitations in that they included only small numbers of healthy participants and failed to control for or monitor the overall diet of their subjects. In addition, they yielded inconclusive results [11,12], presumably by not taking into account the fact that actual diets consist of complex mixtures of different foods. Therefore, to examine the role of nutrients in determining muscle strength thoroughly, this study used a 24-h recall dietary questionnaire based on typical Korean food items. The inconsistent findings of previous studies prompted us to design a study that included a representative population, based on a large-scale and reliable dataset from a national survey.

This study was performed to evaluate the association between HGS and dietary intake in free-living older adults, after adjusting for multiple confounding factors, using a large-scale nationally representative dataset. Our findings might contribute to improvement of evidence-based nutritional advice for physically healthy aging.

## 2. Materials and Methods 

### 2.1. Data Resources

**Figure 1:**
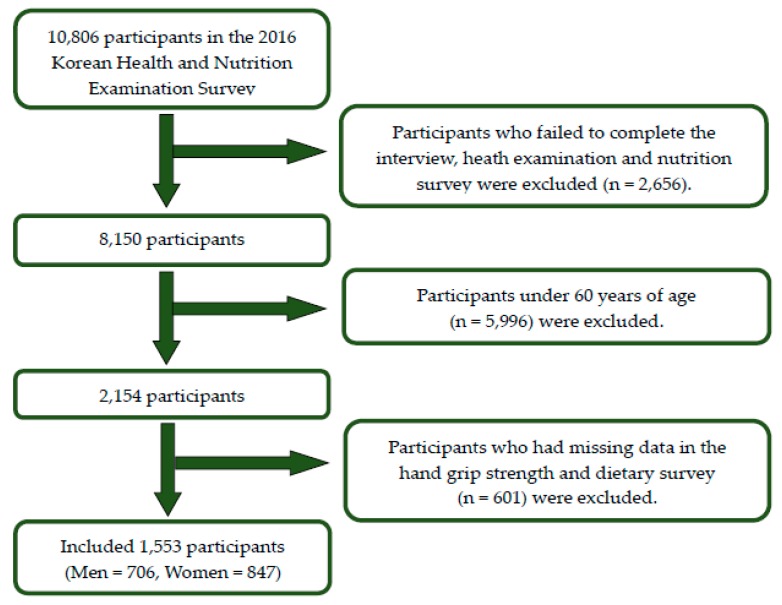
Flow diagram of the study participants.

The data used for this study were collected from the 7th Korean National Health and Nutrition Examination Survey (KNHANES VII-1) in 2016. KNHANES is an ongoing, nationwide cross-sectional survey conducted by the Korea Center for Disease Control and Prevention (KCDC) to assess the health status of Koreans, and to monitor trends in health risk factors and prevalence of major chronic disease in Korea since 1998 [13]. KNHANES includes a complex, stratified, multistage sample. The study targeted households based on geographical area, sex, and age group. Informed consent was obtained from all subjects included in the study. Of the total of 10,806 subjects in the 2016 KNHANES, 8150 participated in the interview, health examination, and nutrition survey, representing a response rate of 75.4%. This study included those in older adults (≥60 years old) (Figure 1) whose data included analytical variables such as HGS and diet (via questionnaire). Ultimately, 1553 participants (706 men, 847 women) were included in the statistical analysis. KNHANES received approval from the institutional review board of the KCDC. All participants in the survey provided written informed consent.

### 2.2. Measures

The interviews and health examinations were performed by trained interviewers and medical staff using calibrated equipment according to a standardized protocol. The interviewer collected data on demographic characteristics, including living situation, current medical conditions (hypertension, diabetes, dyslipidemia, cerebrovascular accidents, myocardial infarction and angina, osteoarthritis, and osteoporosis), socioeconomic status, and education level. The self-administered questionnaire covered smoking status, alcohol use, and physical activity. A current cigarette smoker was defined as an adult who had smoked at least 100 cigarettes in their lifetime and currently smoked cigarettes [14]. A drinker was defined as drinking, with an average of seven cups for men and five or more for women, more than two times a week [15]. Physical inactivity was defined as not conducting moderate to vigorous physical activities for 150 min or more per week [16]. Health examinations were conducted in a mobile examination center, and included a physical examination, anthropometric measurements, and laboratory tests (blood and urine). Height and weight were measured to the nearest 0.1 cm and 0.1 kg, respectively. Body mass index (BMI) was calculated as weight (kg) divided by height squared (m^2^). Waist circumference was measured at the end of a normal breath using a standard protocol. Systolic blood pressure (BP) was measured using an automated BP measurement device. Blood and urine samples were subsequently analyzed at a certified laboratory. The laboratory data quality control program monitored laboratory performance to ensure that the data met the required standard of accuracy [13]. Hemoglobin was measured by the SLS hemoglobin detection method using an XN-9000™ (Sysmex Corporation, Kobe, Japan). Liver/renal function, fasting glucose, and uric acid were measured using a Hitachi Automatic Analyzer 7600-210 (Hitachi, Tokyo, Japan). Hemoglobin A1c (HbA1c) was measured by high-performance liquid chromatography (HPLC) (Tosoh G8; Tosoh, Tokyo, Japan). Total cholesterol (TC) and triglyceride levels were measured using enzymatic methods. High-sensitivity C-reactive protein (hs-CRP) was measured using immunoturbidimetry (Cobas; Roche, Penzberg, Germany).

### 2.3. Handgrip Strength

HGS was measured using a digital grip strength dynamometer (T.K.K 5401; Takei, Niigata, Japan). Both hands were measured alternately three times for a total of six measurements, and the maximum value was taken as the final grip strength [17]. Patients were excluded if they had functional limitations that made it difficult to measure HGS, a history of wrist surgery within the previous 3 months, or a history of wrist discomfort or pain. For measurement of grip strength, the subjects were instructed to face forward while standing upright, straighten the shoulders and allow both arms to hang straight down naturally, with no flexion or extension of the wrist and elbow, with both feet at the width of the pelvis, and to maintain this posture during grip strength measurement. Final HGS was divided into five groups: lowest quintile, lowest 10%; second quintile, next 22%; middle quintile, next 36%; fourth quintile, next 22%; fifth quintile, top 10%. 

### 2.4. Dietary Intake

Dietary intake was assessed by the 24-h recall method, which is an open-label nutritional survey method of estimating food intake. Dietary survey data obtained using the 24-h recall method can reflect the recent dietary intake of individuals and groups. Trained interviewers performed computer-assisted personal interviews (CAPIs) to investigate all food products ingested by the study subjects during the previous 24 h, together with dietary information (time, location, type of food, amount, cooking method).

### 2.5. Statistical Methods

Weighted complex sampling analysis was used for all data analyses. The demographic and clinical characteristics of the study participants are presented as means and standard error. The chi-squared or *t* test was used to compare demographic characteristics between males and females. HGS, laboratory data, and nutritional intake were compared according to age group using a general linear model. Correlation coefficients are presented for the correlations between HGS and nutritional intakes. To take into consideration the results that men and women have significantly different HGS and patterns of dietary intake, we divided subjects into two groups according to gender and analyzed data separately. Generalized linear regression analysis was conducted to determine the individual effects of demographic, clinical characteristics and nutritional intakes on HGS, unadjusted, adjusted for age. Subsequently, variables shown as *p* < 0.05 in the regression model were included as covariates in the final multivariable linear regression analysis (men; low household income, education of less than 10 years, hypertension, dyslipidemia, BMI, alcohol drinking, physical inactivity, total energy intake, women; low household income, education of less than 10 years, total energy intake) as well as covariates considered clinical important (presence of spouse, DM, current smoking) to examine the associations between HGS and each nutrient. Statistical analyses were performed using IBM^®^ SPSS^®^ Statistics for Windows software (ver. 23.0; IBM Corp., Armonk, NY, USA). In all analyses, *p* < 0.05 was deemed to indicate statistical significance.

## 3. Results

### 3.1. Demographic and Clinical Characteristics of Study Subjects

In total of 1553 subjects participated in the study, consisting of 706 (45.5%) men and 847 (54.57%) women with a mean age of 70.1 years. The demographic and clinical characteristics of the study population are shown in Table 1. As shown in the table, 70.9% of the participants had a spouse and 32.4% had over 10 years of education. Women had a lower household income, educational level; a higher rate of dyslipidemia, osteoarthritis and osteoporosis; and were less likely to live with their spouse, be current smokers or consume alcohol than men. The mean BMI and systolic BP in women were significantly higher than that in men.

### 3.2. Handgrip Strength, Laboratory Values, and Nutritional Intake of Study Subjects

Table 2 shows the HGS of the study population. Mean HGSs differed significantly between the 60–64 years and >65 years age groups in both sexes. There were significant differences in laboratory profiles between the 60–64 years and >65 years age groups in both sexes, with males in the younger group having higher levels of hemoglobin, TC, and triglyceride, while females in the 60–64 years age group had higher levels of hemoglobin, alanine aminotransferase (ALT), and TC compared to those aged >65 years. 

Compared to the 60–64 years age group, the >65 years age group had higher levels of blood urea nitrogen and creatinine. All nutrients were consumed more by the 60–64 years age group compared to the >65 years age group in both men and women, although there were no significant differences in intakes of vitamin A, carotene and vitamin C in men, carotene in women.

### 3.3. Associations between Handgrip Strength and Nutritional Intakes

The correlations between HGS and nutritional intakes are shown in Table 3. Total energy, protein, PUFA, dietary fiber, calcium, iron, potassium, vitamin A, carotene, thiamine, niacin, and vitamin C were significantly correlated with HGS. Hemoglobin, ALT, creatinine, FPG, TC, and uric acid levels were also significantly correlated with HGS. In men, low house income, education of less than 10 years, hypertension, dyslipidemia, BMI, alcohol drinking, physical inactivity, and total energy intake were significantly associated with HGS after adjusting for age (Table 4). In women, low household income, education of less than 10 years, BMI, and total energy intake were significantly associated with HGS after adjusting for age (Table 4). In multivariable regression analysis (Table 5), dietary intake of PUFA (*β* = 0.083), and vitamin C (*β* = 0.001) had significant associations with HGS after adjusting for covariates in women. In men, dietary fiber (*β* = 0.071) and vitamin C (*β* = 0.006) were positively associated with HGS.

## 4. Discussion

The results of this study indicate that community-dwelling older men and women with relatively high amounts of PUFA, dietary fiber, and vitamin C in their diet had an elevated likelihood of higher HGS even after adjusting for total calorie intake per day, body weight, and physical activity. To our knowledge, there have been no previous reports of associations between PUFA, dietary fiber, and muscle strength in older adults based on a large-scale dataset representative of the general population. We also confirmed that HGS was closely related to BMI, house income, and education level in older adults (additionally, physical inactivity in men) in addition to uncorrectable factors such as age.

In addition, the mean HGS values in our male and female subjects between 60 and 64 years of age (40.1 and 23.9 kg, respectively) were similar to the reference values for HGS in the Korean population with a mean age of 38.3 years (39.5 and 24.2 kg, respectively) [18]. The majority of older adults in Korea seemed to have adequate dietary intake based on the 2015 reference values for Koreans [19], with the exception of calcium, which was insufficient in both men and women in all age groups.

In this study, we chose HGS as a determinant of muscle strength in older adults. HGS, a commonly used indicator of muscle function, can also be used as a rapid, cost-effective method for nutritional assessment; many reports have indicated that it is significantly correlated with nutritional status [20,21]. These previous studies indicated that people at nutritional risk have lower HGS. This may be because poor nutrient intake can result in reduced protein synthesis, which causes muscle fiber atrophy and decreased muscle mass, in turn leading to impaired muscle function.

There have been few studies regarding the effects of dietary PUFA on muscle strength in older adults using large-scale data sufficient to represent the general population. To improve cardiac and cognitive function, and lower BP, PUFA is helpful. A recent review indicated that PUFA administration predicts improved physical endurance, anti-inflammatory and antioxidant responses, and resistance to delayed-onset muscle soreness in young athletes [22]. The mechanisms underlying these favorable effects of PUFA on muscle strength have yet to be elucidated. However, we assume that alterations in both anabolic and catabolic pathways may be involved. A previous study of isolated mouse muscles indicated that PUFA attenuated muscle protein breakdown [23], and a human study found that fish oil-derived PUFA increased the rate of muscle protein synthesis [24]. The effects of PUFA on muscle strength may also be related to their beneficial impact on muscle lipid and mitochondrial function, which are major determinants of muscle function [25]. Several studies performed using cell culture and animal models implied that PUFAs derived from fish oil stimulate mitochondrial biogenesis and content in muscle, and reduce both total muscle and intramyocellular triglyceride contents [26,27]. Furthermore, the beneficial effects of PUFA on muscle strength may be mediated by the neuroprotective and excitatory properties of motor neurons [28]. A study of 60 healthy men and women aged 60–85 years indicated that PUFA therapy (*n* = 40) for six months significantly increased HGS by 2.3 kg (95% confidence interval: 0.8–3.7 kg), and one-repetition maximum muscle strength by 4.0% (95% CI: 0.8–7.3%) [29]. The improvements in muscle mass and function induced by PUFA in this study were of the same magnitude, or greater, than those reported for anabolic agents, such as testosterone and growth hormone [30], or dehydroepiandrosterone [31], but less pronounced than those associated with exercise training [28]; this implies that fish oil-derived PUFA supplementation should be considered as a potential therapeutic option to slow, and possibly prevent, age-related decline in physical function. Unfortunately, there have been no reports regarding optimal dosages and durations of PUFA treatment for older adults. Therefore, further studies are required, taking into consideration factors such as age, sex, comorbidities, etc.

This study also confirmed a positive correlation between HGS and vitamin C levels. Skeletal muscle continues to produce oxidant species, such as reactive oxygen species (ROS), which act as signaling molecules in the normal processes of repair and regeneration and stimulate mitochondrial biogenesis during exercise, in concert with antioxidant mechanisms [32]. Any imbalance due to an increase in oxidant level and/or a decrease in antioxidative effects results in loss of normal redox equilibrium in cells, a condition known as oxidative stress, which can injure various cellular organelles, proteins, lipids, and membranes, and eventually affect muscle function [33]. Numerous in vivo and in vitro studies have shown that although different mechanisms are involved in the development of impaired muscle function with aging, they are similar in terms of being mediated by oxidative stress and can generate harmful impacts or accelerate muscle damage [32,33,34]. As vitamin C is best known for its antioxidative effects, which protect various tissues against oxidative stress, it is not surprising that it was positively correlated with HGS in the present study. Consistent with our observations, Bobeuf et al. reported tentative evidence that vitamin C (1000 mg/day) supplementation along with strength training in older adults untrained participants facilitated muscle gain over a period of six months [35]. This study implied that older adults can be expected to show more positive responses to antioxidants than their younger counterparts due to age-related changes in the redox conditions of muscles. Indeed, in an animal study, Ryan et al. demonstrated that supplementation with vitamin C and E improved muscle work capacity in older, but not young, rats [34]. Although it is generally agreed that vitamin C can help protect cell structures, such as the sarcoplasmic reticulum, from oxidative stress and free radical injury, few studies have examined the minimum or ideal doses of vitamin C to prevent age-related deterioration of muscle strength, making it difficult to draw any firm conclusions at present.

We suggest that the favorable association of dietary fiber with HSG may be attributable to both antihyperglycemic and antilipidemic effects. Dietary fiber has two major physiological effects, i.e., blunting the increase in postprandial plasma glucose and insulin by slowing gastric emptying and glucose diffusion into the small intestine, and lowering cholesterol levels [36]. These processes reduce glucose absorption after a meal and decrease adiposity. Recent animal studies implied that dietary fiber may increase fatty acid oxidation, which causes changes in adiposity, by showing that mice fed a high-glycemic diet have blunted fatty acid oxidation in contrast to mice fed a low-glycemic diet [37]. These findings indicate that dietary fiber can help maintain lean body mass and decrease adiposity, by increasing mitochondrial biogenesis and fatty acid oxidation in skeletal muscle. Thus, dietary fiber may be a useful dietary component for older adults.

In this study, there was no association between dietary protein and HSG after adjusting for covariates. This result is consistent with those of a few clinical trials, as well as other cross-sectional studies, which failed to show significant improvement in physical performance with addition of high-quality protein to the diet or supplementation during intervention periods ranging from three months to two years [38,39] not only in healthy older women but also in sarcopenic men and women. Data on the body composition of the subjects were not available in the present study, since KNHANES did not measure the body composition of the older participants. Deterioration of muscle strength is related to a decrease in muscle mass in older adults [40]. However, this relationship is not necessarily linear, with the decline in strength being more rapid than mass loss [40]. In this respect, muscle mass has been reported to predict only a small precentage (<5%) of the decrease in muscle strength associated with aging [41]. 

Generally, men have greater HGS than women in all age groups [18,42], and our results also reflected this sex difference. We found that in Korea the BMI of older men was lower than that of women. Previous studies involving all age groups reported positive correlations of HGS with BMI in both men and women. Based on our results, BMI may have less impact on HGS in older adults than in younger age groups. 

In this study, dietary intake data of the subjects were obtained from the KNHANES VII-1 survey using the 24-h dietary recall. The amount of total energy and nutrients consumed were calculated using a food composition table published by the Rural Development Administration of Korea and its validity and reliability are acceptable [43,44,45]. The 24-h recall has been widely used to estimate dietary patterns in large epidemiological studies. This is a structured interview designed to obtain information about foods and beverages consumed by a respondent in the past 24 h. In contrast to food frequency questionnaire (FFQs), its open-ended response structure allows the respondent to provide a comprehensive and detailed report of all foods. Food models, pictures, and other visual aids are used to help respondents judge and describe portion size and improve accuracy. However, since the 24-h dietary recall is affected by day-to-day variation, it may not represent the usual intake of the subjects. In addition, it requires well trained interviewers and can be relatively expensive and time-consuming compared to the FFQs method [46]. 

This study had a number of strengths. One of major strengths was the large-scale, nationally representative sampling of the general Korean older population. Second, we used the most reliable data available, including a variety of health-related questions and tests, which allowed us to control for various potential confounding factors associated with HSG and nutritional status. Moreover, to our knowledge, this is the first study in which PUFA has been found to be related to muscle strength in older Koreans. However, the study also had some limitations. First, it used a cross-sectional design, in which all data were collected simultaneously such that the causative relationship between PUFA and HGS is uncertain. Second, the correlations of PUFA, vitamin C, and dietary fiber with HGS were so weak that these findings may not be clinically significant. However, as it is difficult to demonstrate the effects of nutrition on muscle strength in human studies, because they can be influenced by various factors over the entire lives of the subjects, these weak correlations can be considered clinically important. In addition, dietary evaluation in this study was restricted to single-day recall. Therefore, we were unable to distinguish between the effects of consumption of nutrients in foods versus as supplements, including the effects on the maintenance of homeostasis. 

In conclusion, we found positive associations between HSG and PUFA, dietary fiber, and vitamin C intakes in Korean free-living older adults. These observations should prompt future interventional studies to determine important nutrients and ideal doses to ameliorate age-related muscle strength deterioration. 

## 5. Conclusions

Our results suggest that PUFA, dietary fiber and vitamin C might be beneficial for maintaining physical function among older adults. Given that these nutrients are readily available by consuming healthy food, and relatively inexpensive and safer compared to other anabolic agents such as testosterone and growth hormone, a diet that is rich in PUFA, dietary fiber and vitamin C could first be recommended as a potential therapeutic option for age-related decline in muscle strength in clinical settings.

## Figures and Tables

**Table 1:** Demographic characteristics of study subjects in the 2016 KNHANES VII-1.

Variables	Men (*n* = 706)	Women (*n* = 847)	Total (*n* = 1553)
Demographics			
Age (years)	70.1 ± 6.3	70.0 ± 6.6	70.1 ± 6.4
Low household income (%) *	33.7 ± 2.5	43.4 ± 2.3	39.0 ± 2.0
Presence of spouse (%) *	87.7 ± 1.6	56.8 ± 2.1	70.9 ± 1.6
Education ≥ 10 years (%) *	46.1 ± 4.3	20.6 ± 2.8	32.4 ± 2.8
Current medical history			
Hypertension (%)	52.7 ± 2.3	46.4 ± 2.0	49.3 ± 1.5
Diabetes mellitus (%)	20.0 ± 1.8	19.2 ± 1.7	19.6 ± 1.3
Dyslipidemia (%) *	18.7 ± 1.8	31.1 ± 2.1	25.4 ± 1.3
CVA (%)	3.8 ± 0.7	2.0 ± 0.5	2.9 ± 0.5
MI & angina (%)	8.2 ± 1.1	5.3 ± 0.9	6.7 ± 0.6
Osteoarthritis (%) *	9.5 ± 1.3	31.2 ± 1.6	21.2 ± 1.1
Osteoporosis (%) *	1.3 ± 0.5	31.5 ± 1.9	17.7 ± 1.1
Anthropometric measurements			
BMI (kg/m^2^) *	23.8 ± 0.1	24.5 ± 0.1	24.2 ± 0.1
AC (cm) *	86.6 ± 0.4	84.6 ± 0.4	85.6 ± 0.3
Systolic BP (mm Hg) *	126.1 ± 0.7	128.2 ± 0.7	127.2 ± 0.5
Health behaviors			
Current smoking (%) *	19.8 ± 1.8	3.6 ± 1.2	11.0 ± 1.2
Dringking (%) *	61.9 ± 2.2	20.0 ± 1.6	39.2 ± 1.6
Physical inactivity (%) *	55.1 ± 2.2	67.3 ± 2.4	61.7 ± 1.7

* *p* < 0.05 by chi-squared test or *t*-test. Data are presented as estimated means ± standard errors. CVA, cerebrovascular accident; MI, myocardial infarction; AC, abdominal circumference; BMI, body mass index; BP, blood pressure; low income level—people in the lowest quartile of household income; presence of spouse—people who currently live with his or her spouse; current smoking—adults who had smoked at least 100 cigarettes in their lifetime and currently smoked cigarettes every day (daily) or on some days (nondaily); drinking—drinking at least twice per week, with an average of 7 cups (for women, 5 cups) or more; physical inactivity—people who do not conduct moderate to vigorous physical activities for 150 min or more per week.

**Table 2:** Handgrip strength, laboratory data, and nutritional status of study subjects.

Variable	Men (*n* = 706)		*p*	Women (*n* = 847)		*p*
	Age 60–64 (*n* = 173)	Age ≥65 (*n* = 533)		Age 60–64 (*n* = 242)	Age ≥65 (*n* = 605)	
Handgrip strength (kg)	40.1 ± 0.6	34.2 ± 0.4	<0.001	23.9 ± 0.3	20.1 ± 0.2	<0.001
Lowest quintile	27.2 ± 0.3	19.0 ± 0.5		15.6 ± 0.5	14.0 ± 0.2	
Second quintile	30.8 ± 0.4	25.7 ± 0.2		19.3 ± 0.1	19.2 ± 0.1	
Middle quintile	35.8 ± 0.3	31.3 ± 0.2		22.9 ± 0.1	22.6 ± 0.1	
Fourth quintile	40.9 ± 0.2	36.6 ± 0.1		26.1 ± 0.1	26.2 ± 0.1	
Fifth quintile	47.6 ± 0.7	43.0 ± 0.4		29.6 ± 0.3	29.2 ± 0.2	
Laboratory profiles						
Hemoglobin (g/dL)	15.1 ± 0.1	14.5 ± 0.1	<0.001	13.3 ± 0.1	12.9 ± 0.1	<0.001
AST (IU/L)	24.8 ± 1.2	24.3 ± 0.6	>0.05	24.7 ± 0.9	23.4 ± 0.5	>0.05
ALT (IU/L)	23.5 ± 0.8	21.7 ± 0.7	>0.05	24.1 ± 1.4	19.0 ± 0.6	<0.01
BUN (mg/dL)	15.9 ± 0.4	17.3 ± 0.3	<0.01	15.4 ± 0.3	16.4 ± 0.3	<0.05
Creatinine (mg/dL)	0.95 ± 0.01	1.02 ± 0.01	<0.001	0.72 ± 0.01	0.79 ± 0.01	<0.001
Fasting glucose (mg/dL)	112.6 ± 2.6	110.2 ± 1.5	>0.05	102.9 ± 1.6	104.9 ± 1.0	>0.05
HbA1c (%)	5.93 ± 0.06	6.02 ± 0.05	>0.05	5.94 ± 0.05	6.00 ± 0.03	>0.05
Total cholesterol (mg/dL)	193.6 ± 3.5	181.1 ± 1.9	<0.01	202.7 ± 2.6	190.1 ± 1.6	<0.001
Triglycerides (mg/dL)	181.4 ± 16.9	138.6 ± 4.1	<0.05	141.3 ± 7.3	142.2 ± 4.3	>0.05
High-sensitivity CRP (mg/L)	1.52 ± 0.23	1.6 ± 0.1	>0.05	1.20 ± 0.16	1.48 ± 0.15	>0.05
Uric acid (mg/dL)	5.6 ± 0.1	5.6 ± 0.1	>0.05	4.1 ± 0.1	4.6 ± 0.1	>0.05
Nutritional intakes						
Total energy (Cal/day)	2234.3 ± 74.0	1935.0 ± 38.3	<0.001	1646.6 ± 46.5	1365.4 ± 26.4	<0.001
Protein/weight (g/kg/day)	1.18 ± 0.06	0.96 ± 0.02	<0.01	1.01 ± 0.05	0.78 ± 0.02	<0.001
PUFA (g/day)	10.9 ± 0.7	8.3 ± 0.4	<0.01	8.4 ± 0.5	6.1 ± 0.2	<0.001
Fiber (g/day)	28.3 ± 1.3	25.2 ± 0.6	<0.05	23.0 ± 0.8	19.8 ± 0.6	<0.01
Calcium (mg/day)	589.3 ± 30.5	452.5 ± 15.2	<0.001	421.9 ± 16.3	330.5 ± 8.7	<0.001
Fe (mg/day)	21.0 ± 1.7	17.6 ± 0.5	<0.05	16.7 ± 1.1	12.6 ± 0.3	<0.01
Potassium (mg/day)	3514.5 ± 142.2	2992.8 ± 87.8	<0.01	2954.1 ± 96.4	2327.9 ± 64.6	<0.001
Vitamin A (µgRE/day)	793.1 ± 64.1	669.3 ± 37.0	>0.05	576.3 ± 40.4	468.7 ± 23.4	<0.05
Carotene (µg/day)	3966.2 ± 361.2	3540.4 ± 210.2	>0.05	2882.5 ± 217.1	2449.9 ± 136.8	>0.05
Thiamine (mg/day)	2.3 ± 0.1	1.9 ± 0.0	<0.01	1.7 ± 0.1	1.4 ± 0.04	<0.001
Niacin (mg/day)	17.6 ± 1.0	14.2 ± 0.4	<0.01	13.9 ± 0.6	10.0 ± 0.2	<0.001
Vitamin C (mg/day)	126.1 ± 12.1	110.6 ± 6.6	>0.05	149.2 ± 11.4	99.4 ± 5.5	<0.001

*p* < 0.05 by chi-squared test or *t*-test. Data are presented as estimated means ± standard errors. AST, aspartate transferase; ALT, alanine transferase; BUN, blood urea nitrogen; CRP, C-reactive protein; PUFA, polyunsaturated fatty acid.

**Table 3:** Correlations of handgrip strength with nutritional intakes in study subjects.

Variables	HGS	Hb	AST	ALT	BUN	Cr	FPG	HbA1c	TC	TG	hs-CRP	UA
HGS		0.502 *	0.042	0.130 *	−0.004	0.239 *	0.052 ^†^	−0.019	−0.059 ^†^	0.032	−0.044	0.296 *
Total energy	0.411 *	0.256 *	0.004	0.032	−0.022	0.050	−0.016	−0.057	−0.001	0.030	−0.003	0.112 *
Protein/weight	0.217 *	0.109 *	−0.037	−0.033	−0.018	−0.029	−0.073 *	−0.085 *	0.020	−0.048	−0.002	−0.016
PUFA	0.269 *	0.127 *	−0.038	0.031	0.011	0.018	−0.017	−0.022	−0.028	−0.017	−0.041	0.048
Fiber	0.272 *	0.166 *	−0.019	0.030	0.001	−0.007	0.001	−0.017	−0.004	0.007	−0.039	0.041
Calcium	0.296 *	0.190 *	−0.026	0.049	−0.011	0.019	0.000	−0.019	0.036	0.004	−0.033	0.053 ^†^
Fe	0.222 *	0.149 *	−0.014	0.014	−0.011	0.002	−0.008	−0.042	0.013	0.006	−0.016	0.038
Potassium	0.300 *	0.166 *	−0.033	0.028	−0.023	−0.002	−0.044	−0.043	0.037	0.019	−0.042	0.066 ^†^
Vitamin A	0.159 *	0.104 *	0.018	0.042	−0.003	−0.012	0.029	0.012	−0.003	−0.007	−0.037	−0.015
Carotene	0.141 *	0.100 *	0.022	0.039	−0.001	−0.008	0.040	0.019	−0.017	−0.003	−0.033	−0.012
Thiamine	0.364 *	0.213 *	−0.032	0.034	−0.016	0.003	−0.028	−0.055 ^†^	0.012	0.003	−0.007	0.061 ^†^
Niacin	0.343 *	0.196 *	−0.029	0.016	−0.002	0.004	−0.048	−0.058 ^†^	0.014	−0.005	−0.030	0.067 ^†^
Vitamin C	0.098 *	0.056 ^†^	−0.026	0.028	−0.059 ^†^	−0.046	−0.076 *	−0.090 *	0.061 ^†^	−0.023	−0.026	−0.014

* *p* < 0.01 or ^†^ *p* < 0.05 for the correlation coefficient. HGS, handgrip strength; Hb, hemoglobin; AST, aspartate transferase; ALT, alanine transferase; BUN, blood urea nitrogen; Cr, creatinine; FPG, fasting plasma glucose; HbA1c, hemoglobin A1c; TC, total cholesterol; TG, triglyceride; hs-CRP, high-sensitivity C-reactive protein; UA, uric acid; PUFA, polyunsaturated fatty acid.

**Table 4:** Handgrip strength and clinical characteristics, nutritional status.

Variable	Women						Men					
	Unadjusted			Adjusted for Age			Unadjusted			Adjusted for Age		
	*β*	SE	*p*	*β*	SE	*p*	*β*	SE	*p*	*β*	SE	*p*
Age	−0.348	0.024	<0.001				−0.630	0.049	<0.001			
Low household income	−2.416	0.346	<0.001	−0.902	0.329	0.006	−4.381	0.611	<0.001	−1.458	0.663	0.028
Presence of spouse	1.560	0.383	<0.001	0.392	0.329	0.234	2.570	0.940	0.007	−1.414	0.869	0.104
Education < 10 year	−2.152	0.417	<0.001	−0.862	0.389	0.027	−3.510	0.587	<0.001	−2.594	0.505	<0.001
Hypertension	−1.155	0.363	0.002	−0.095	0.333	0.776	0.670	0.701	0.340	0.236	0.579	0.400
DM	−1.256	0.502	0.013	−0.265	0.470	0.574	−0.785	0.703	0.265	−0.839	0.654	0.200
Dyslipidemia	−1.200	0.388	0.002	−0.478	0.336	0.156	−3.685	0.704	<0.001	−2.578	0.671	<0.001
BMI (kg/m^2^)	0.165	0.059	0.006	0.147	0.049	0.003	0.758	0.117	<0.001	0.555	0.98	<0.001
Current smoking	1.019	1.369	0.457	0.797	0.897	0.375	0.319	0.856	0.710	−0.254	0.735	0.730
Drinking	−1.138	0.485	0.019	−0.307	0.404	0.448	−2.961	0.713	<0.001	−1.174	0.592	0.048
Physical inactivity	−1.548	0.353	<0.001	−0.557	0.360	0.122	−2.831	0.605	<0.001	−1.556	0.560	0.001
Total energy (Cal/day)	0.002	0.000	<0.001	0.001	0.000	0.002	0.002	0.000	<0.001	0.001	0.000	0.011
Protein/weight (g/kg/day)	1.706	0.364	<0.001	0.405	0.317	0.203	1.787	0.636	0.005	0.334	0.498	0.502
PUFA (g/day)	0.216	0.026	<0.001	0.124	0.024	<0.001	0.200	0.051	<0.001	0.128	0.043	0.003
Fiber (g/day)	0.087	0.014	<0.001	0.043	0.013	0.001	0.122	0.022	<0.001	0.073	0.019	<0.001
Calcium (mg/day)	0.004	0.001	<0.001	0.001	0.001	0.156	0.005	0.001	<0.001	0.003	0.001	0.004
Fe (mg/day)	0.069	0.026	0.007	0.025	0.012	0.039	0.066	0.038	0.085	0.024	0.028	0.388
Potassium (mg/day)	0.001	0.000	<0.001	0.001	0.000	0.005	0.001	0.000	<0.001	0.001	0.000	<0.001
Vitamin A (µgRE/day)	0.001	0.000	0.010	0.001	0.000	0.290	0.001	0.000	<0.048	0.000	0.000	0.297
Carotene (µg/day)	0.001	0.000	0.037	0.001	0.000	0.340	0.001	0.000	0.107	0.000	0.000	0.340
Thiamine (mg/day)	1.689	0.241	<0.001	0.785	0.219	<0.001	1.673	0.342	<0.001	0.565	0.321	0.079
Niacin (mg/day)	0.194	0.033	0.001	0.081	0.028	0.003	0.207	0.061	0.001	0.099	0.054	0.065
Vitamin C (mg/day)	0.009	0.001	<0.001	0.005	0.001	<0.001	0.010	0.003	0.002	0.007	0.003	0.007

*p*-values were obtained using generalized regression analysis in a complex sample design. *β*, standardized coefficients; BMI, Body mass index; DM, diabetes mellitus; PUFA, polyunsaturated fatty acid; SE, standard error; low income level—people in the lowest quartile of household income; presence of spouse—people who currently live with his or her spouse; Current smoking—adults who had smoked at least 100 cigarettes in their lifetime and currently smoked cigarettes every day (daily) or on some days (nondaily); drinking—drinking at least twice per week, with an average of 7 cups (for women, 5 cups) or more; physical inactivity—people who do not conduct moderate to vigorous physical activities for 150 min or more per week.

**Table 5:** Associations between handgrip strength and nutritional variables.

Nutritional Intake	Men				Women			
	*β*	SE	95% CI	*p*	*β*	SE	95% CI	*p*
PUFA (g/day)	0.027	0.054	−0.080, 0.133	0.624	0.083	0.029	0.025, 0.141	0.005
Dietary fiber (g/day)	0.071	0.023	0.026, 0.116	0.002	0.019	0.016	−0.012, 0.049	0.235
Calcium (mg/day)	0.001	0.001	−0.001, 0.003	0.244	−0.001	0.001	−0.003, 0.001	0.396
Fe (mg/day)	0.013	0.022	−0.030, 0.057	0.546	0.004	0.010	−0.015, 0.023	0.681
Potassium (mg/day)	0.0001	0.000	0.000, 0.001	0.026	0.000	0.000	0.000, 0.589	0.556
Thiamine (mg/day)	−0.142	0.447	−1.020, 0.736	0.751	0.657	0.372	−0.073, 1.388	0.078
Niacin (mg/day)	0.041	0.088	−0.131, 0.214	0.636	0.025	0.036	−0.046, 0.096	0.491
Vitamin C (mg/day)	0.006	0.002	0.001, 0.011	0.013	0.003	0.001	0.001, 0.005	0.022

*p*-values were obtained using multiple generalized regression analysis in a complex sample design adjusted for age, body mass index, household income, presence of spouse, education level, history of hypertension, diabetes mellitus, and dyslipidemia, smoking status, alcohol consumption, physical inactivity, and total energy intake. *β*—standardized coefficients; BMI—Body mass index; CI—confidence interval; PUFA—polyunsaturated fatty acid; SE—standard error.
